# Arthroscopic Suprapectoral Biceps Tenodesis Below the Groove: A Surgical Technique

**DOI:** 10.1016/j.eats.2025.103707

**Published:** 2025-07-07

**Authors:** Joanne Zhou, Zaamin B. Hussain, Jacob A. Worden, Frank L. Vazquez, Michael B. Gottschalk, Eric R. Wagner

**Affiliations:** a Department of Orthopaedic Surgery, Emory University School of Medicine, Atlanta, Georgia, U.S.A.; b Department of Orthopaedic Surgery, Medical College of Georgia, Augusta, Georgia, U.S.A.

## Abstract

Despite the widespread use of biceps tenodesis, controversy remains surrounding the technique, including the choice between open and arthroscopic approaches, anchor site selection, and inlay versus onlay fixation. We present an arthroscopic technique that places the bony anchor for the long head of the biceps tendon below the bicipital groove with minimal soft tissue disruption, minimal risk for major postoperative complications, and comparable biomechanical outcomes to other techniques. Pearls, pitfalls, advantages, and disadvantages are discussed.

Pathology of the long head of the biceps tendon (LHBT) is a well-known pain generator of the shoulder.^1^ The LHBT originates from the supraglenoid tubercle and superior glenoid labrum,^2^^,^^3^ runs obliquely through the rotator interval into the bicipital groove, and is constrained in the groove by the transverse humeral ligament and portions of the subscapularis and supraspinatus (Fig 1).^4^ The LHBT is densely innervated with sympathetic fibers near its origin, which contributes to the pathogenesis of pain associated with this tendon.^5^

**Fig 1:**
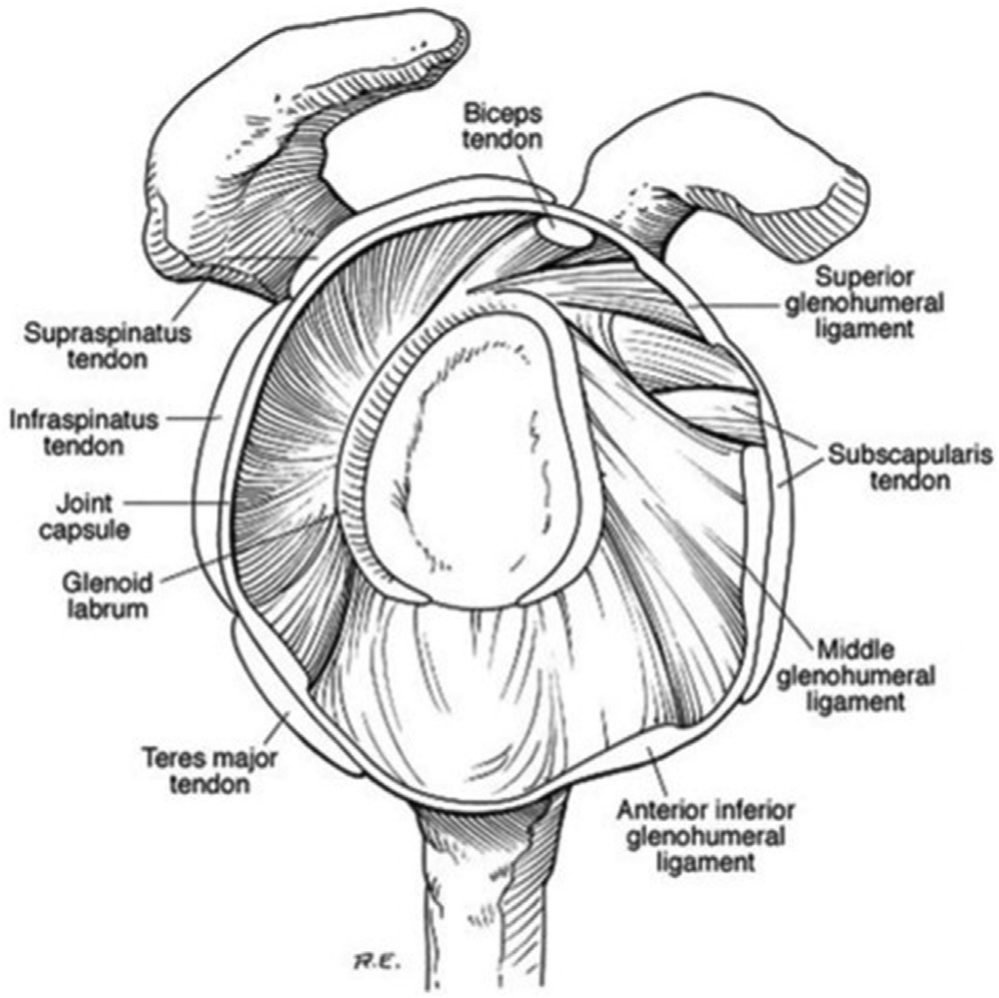
Diagram of arthroscopic shoulder anatomy. Reprinted with permission from Wiley et al. 2005. Wiley WB, Goradia VK, Pearson SE. Arthroscopic capsular plication-shift. *Arthroscopy* 2005;21:119-121.

Biceps tenodesis and tenotomy are common procedures for LHBT tendinopathy, LHBT subluxation, biceps pulley lesions, and SLAP tears.^6^^,^^7^ Although these procedures have similar outcomes,^7^^,^^8^ tenotomy has shorter operative times, decreased cost, and faster recovery,^9^ whereas tenodesis is thought to have increased strength and resistance to load, less muscle cramping, and improved cosmesis by avoiding the “Popeye” deformity.^8^^,^^10^, ^11^, ^12^, ^13^ Biceps tenodesis can be performed open or arthroscopically, in isolation or combination with other procedures, such as rotator cuff repairs and subacromial decompression. Although outcomes are similar, arthroscopic techniques may reduce complications such as infections and nerve injuries by avoiding axillary incisions.^14^, ^15^, ^16^, ^17^ Location of the tenodesis can be above or below the groove, with tenodeses below the groove potentially avoiding postoperative groove pain. Fixation can be onlay with an anchor or button or inlay using a biotenodesis screw. Although similar outcomes have been reported with both of these techniques, the onlay technique avoids the potential “guillotine” effect of the tendon entering the bone at a sharp angle.^18^

We present a simple technique for an arthroscopic onlay biceps tenodesis using a knotless anchor below the bicipital groove.

## Surgical Technique

**Fig 2:**
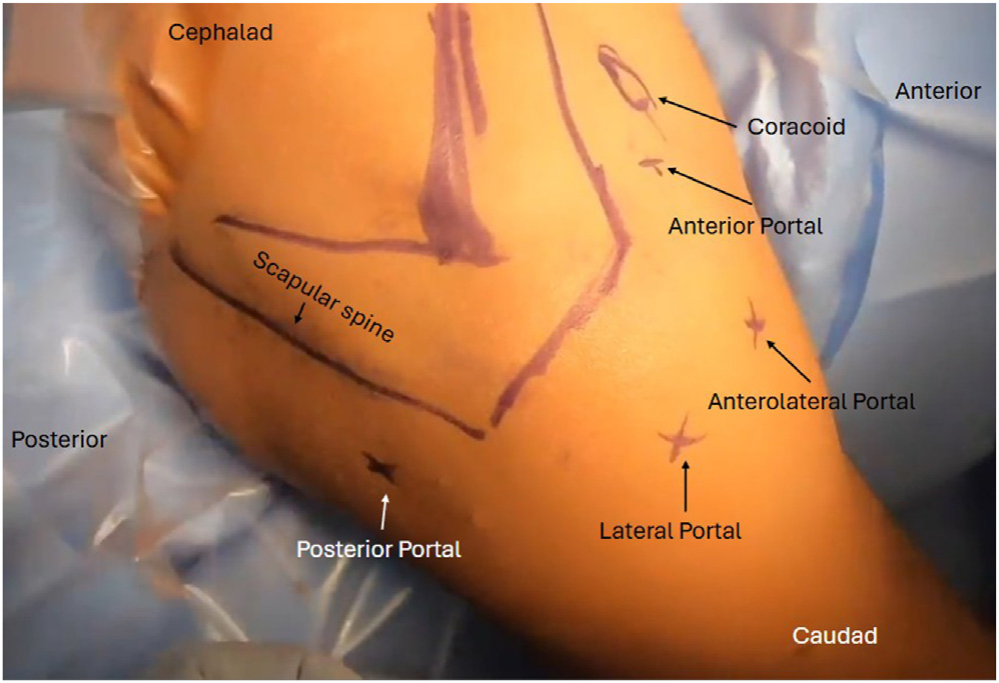
View of patient from the beach-chair position of right shoulder with markings for portals used in arthroscopic biceps tenodesis.

The patient is placed in the beach-chair position, and portals are marked after identifying bony landmarks (Fig 2). Lidocaine with epinephrine is injected into the subacromial space and into the anterior biceps area to assist with hemostasis. A diagnostic arthroscopy is performed utilizing the standard posterior viewing portal, confirming the status of the biceps tendon and superior labrum. Instruments are outlined in Table 1.

**Table 1:** Instruments Needed for Arthroscopic Biceps Tenodesis

Instruments Needed for Arthroscopic Biceps Tenodesis
30° arthroscope (Synergy; Arthrex)
Arthroscopic grasper (Tapered Grasper; Arthrex)
Instrument to complete tenotomy (e.g., arthroscopic cutter)
Local with epinephrine injection
One No. 2 high-strength nonabsorbable suture (e.g., FiberLoop; Arthrex)
Suture anchor (e.g., SwiveLock; Arthrex)
18-Gauge spinal needle

**Fig 3:**
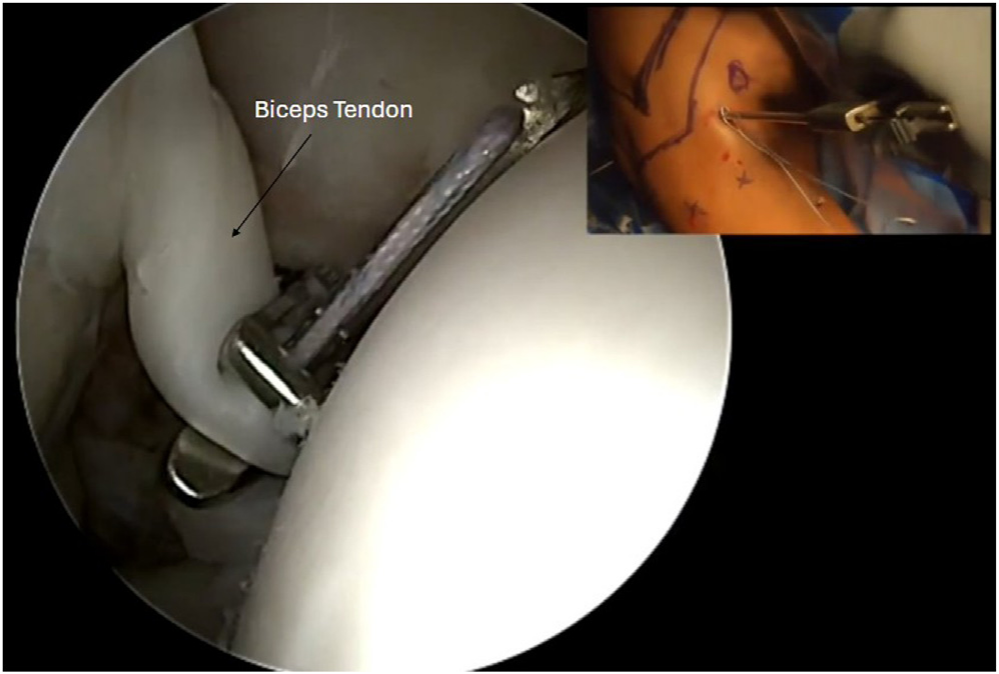
View from the posterior portal of right shoulder using a 30° arthroscope. The biceps tendon is identified and tagged with a looped suture using an arthroscopic suture passer through the anterior portal, at the top of the bicipital groove, approximately 3 cm from its insertion on the supraglenoid tubercle.

The anterior portal is established with the aid of a spinal needle in the rotator interval, and a combination of electrocautery and shaver is used to sufficiently clear out the rotator interval. The biceps tendon is identified and tagged with a looped suture using an arthroscopic suture passer through the anterior portal, at the top of the bicipital groove, approximately 3 cm from its insertion on the supraglenoid tubercle (Fig 3). This tagging location helps to properly tension the biceps below the groove, so it is critical to tag it as close to the top of the groove as possible, adjacent to the margin of the articular cartilage of the humeral head. Arthroscopic scissors are then used to cut the proximal biceps near the supraglenoid tubercle, with countertraction through the tagging suture.

**Fig 4:**
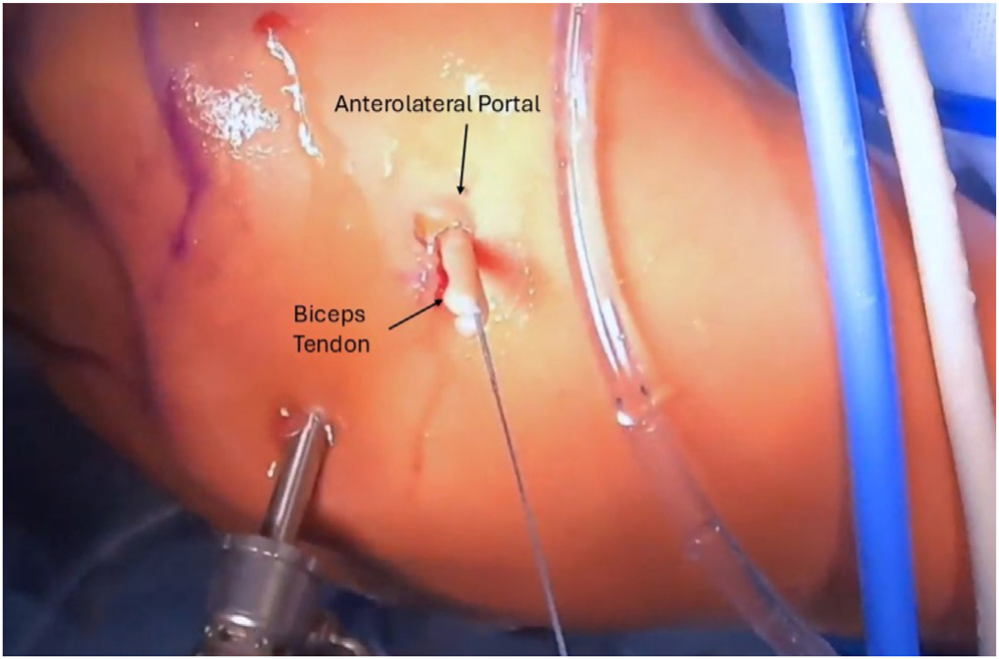
View of patient from the beach-chair position with the biceps tendon retrieved through the anterolateral portal.

**Fig 5:**
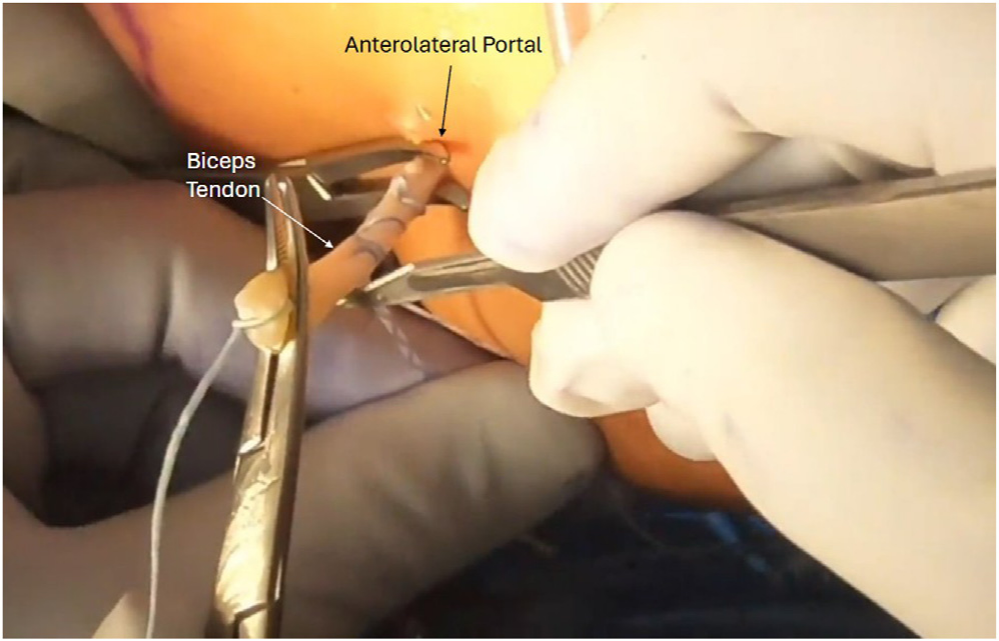
View of patient from the beach-chair position of right shoulder. A clamp is used to grasp the tendon end and a nonabsorbable running locking suture is placed through the tendon with 3 passes, ending 2 to 3 cm proximal to the previous tagged stitch. After this is completed, the tendon edge is trimmed.

**Fig 6:**
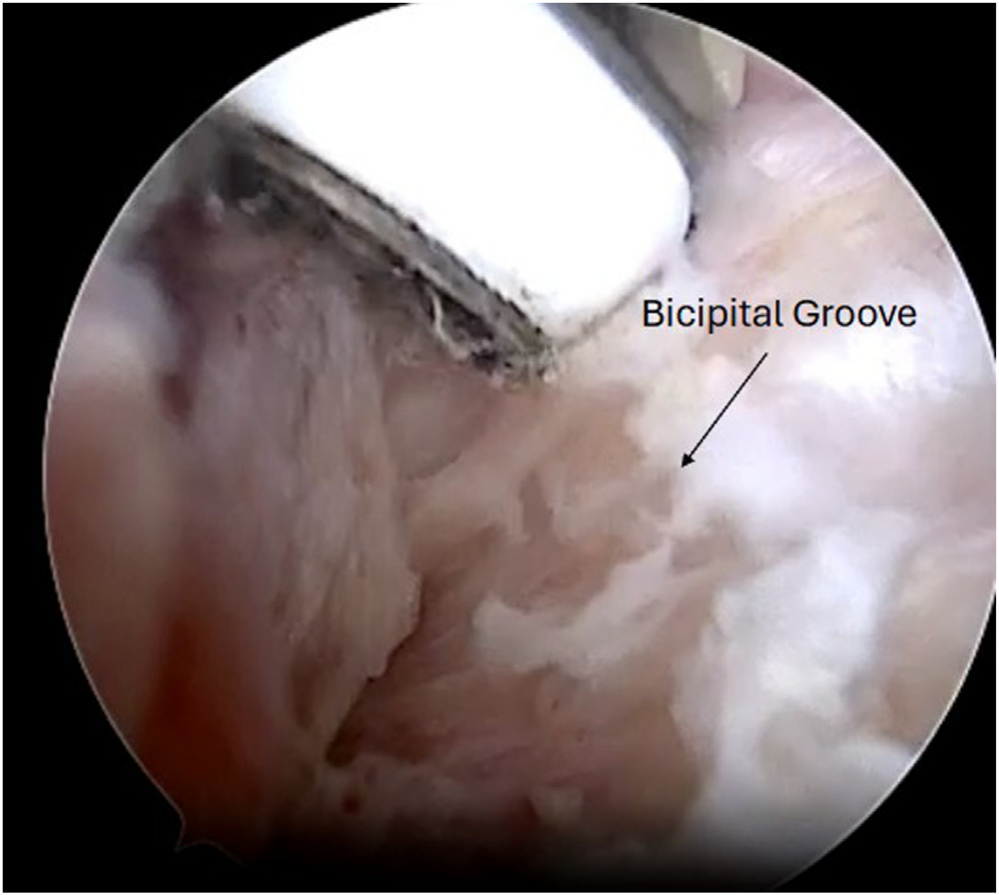
Viewing from the lateral portal of right shoulder with a 30° arthroscope, the footprint for the biceps anchor is cleared below the bicipital groove using electrocautery, and the bone is debrided with a shaver to create an ideal healing environment.

**Fig 7:**
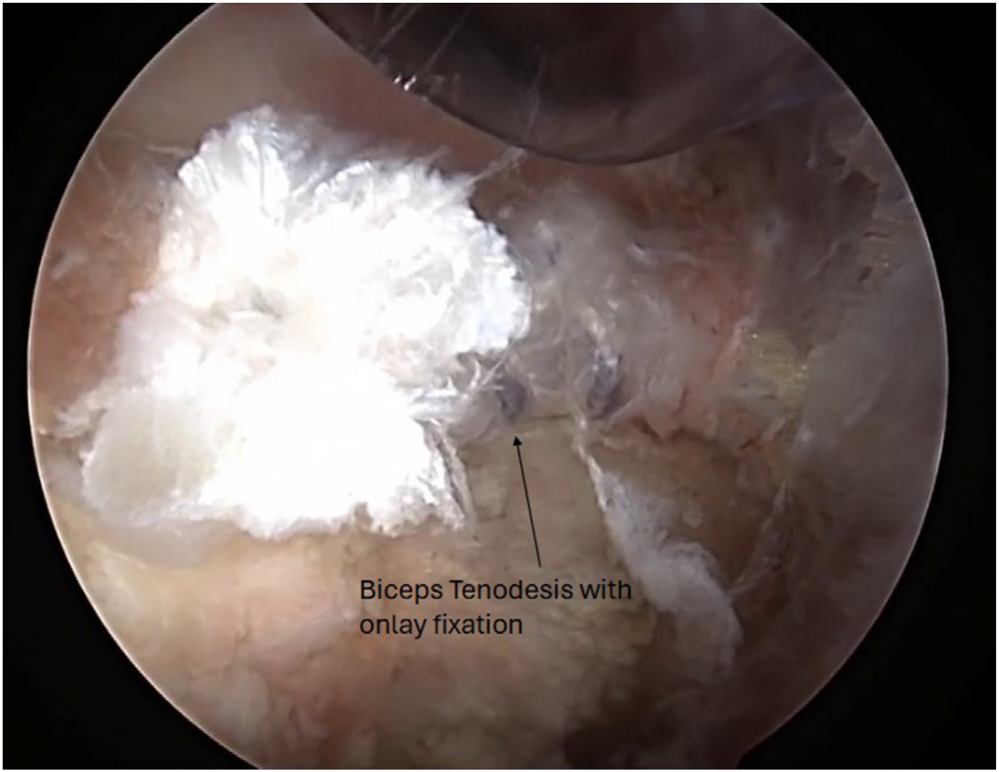
View from anterior portal of right shoulder using 30° arthroscope showing onlay fixation of the biceps tenodesis just below the bicipital groove above the pectoralis major tendon insertion.

Next, the arthroscope is moved in the subacromial space, and subacromial and subdeltoid bursal tissue is debrided until the anterolateral gutter is easily visualized without disrupting the deltoid fascia. The arthroscope is then switched to the lateral portal, with an anterolateral working portal (Fig 2). The lateral aspect of the greater tuberosity is again visualized, and dissection is carried anteriorly using the shaver toward the bicipital groove to expose the biceps tendon. The inferior aspect of the bicipital groove can be identified when crossing blood vessels are seen, a sign of the terminal branches of the “3 sisters” circumflex vessels. It is useful to use electrocautery during the dissection in this area below the groove to obtain hemostasis before exposing the biceps. Flexion and internal rotation improve visualization of the biceps and relieve anterior deltoid tension. The biceps is seen and palpated as it exits the groove under remnants of the transverse ligament, and this is opened up using electrocautery to expose the biceps. It is important not to damage the biceps, but to release it from the fascial bands and ligaments surrounding the biceps superiorly up to the bicipital groove entrance and inferiorly for biceps mobilization. A grasper is used to retrieve the biceps tendon through the anterolateral portal (Fig 4). The initial luggage tag loop is used to estimate the location of the tenodesis stitch, approximately 2 to 3 cm proximal to the tagged suture to create the ideal anatomic tension of the biceps. A clamp is used to grasp the tendon end, and a nonabsorbable running locking suture is placed through the tendon with 3 passes, ending 2 to 3 cm proximal to the previous tagged stitch. After this is completed, the tendon edge is trimmed (Fig 5). The footprint of the anchor insertion is cleared off just below the bicipital groove (Fig 6) using electrocautery, and the bone is debrided with a shaver to create an ideal healing environment. The suture anchor is loaded and placed below the bicipital groove above the pectoralis insertion for onlay fixation (Fig 7). We perform onlay fixation to avoid the “guillotine” amputation risk of the biceps tendon when entering the bone at a sharp angle.^18^ Final arthroscopic images are captured, and portals are closed with dissolvable sutures (Video 1).

### Postoperative Protocol

Patients remain in a shoulder immobilizer for 2 weeks, performing elbow, wrist, and hand motion. After week 2, patients are progressed to active motion in pool therapy, limited to 5 pounds. At 6 weeks, patients are permitted to perform gentle strengthening, and at 12 weeks, patients are permitted to return to most activities. Return-to-sport restrictions are lifted 4 months postsurgery.

## Discussion

We present a simple, reproducible arthroscopic biceps tenodesis technique that anchors the LHBT just below the bicipital groove with the optimal anatomic tension, above the pectoralis major tendon insertion using an onlay technique. See Table 2 for pearls and pitfalls.

**Table 2:** Pearls and Pitfalls for Performing an Arthroscopic Biceps Tenodesis

Pearls	Pitfalls
Inject with lidocaine and epinephrine to decrease bleeding preoperatively	Avoid disrupting the deltoid fascia when performing the bursectomy to aid with visualization
Tage the biceps at the top of the groove 2 to 3 cm from its insertion on the superior labrum	Avoid undue tension on the biceps by placing the anchor for the tenodesis at least 1 cm inferior to the bicipital groove exit distally
Perform an adequate lateral gutter subdeltoid bursectomy with a shaver to have clear visualization of the biceps and transverse ligament	Use electrocautery to release the biceps, given that the branches of the circumflex vessels can lead to poor visualization
Forward flex the arm and internally rotate to open up the space over the biceps and bring it directly under the anterolateral portal	Finishing running locking stitch 2 to 3 cm proximal to the tagging stitch, enabling the optimal tension for the tenodesis

Many approaches for biceps tenodesis have been described, including open, minimally open, and arthroscopic. While open tenodesis uses an intermuscular interval,^19^ its proximity to the axilla increases the risk of wound issues,^20^ hematoma/seroma formation,^20^ infection,^21^ and nerve injury.^20^^,^^22^^,^^23^ Arthroscopic biceps tenodesis allows for the preservation of the surrounding soft tissue and improved visualization, but it can be more technically demanding, with a risk of refractory groove pain if performed at the top of the bicipital groove.^24^^,^^25^ Several studies have noted similar outcomes of arthroscopic versus open tenodesis,^15^^,^^16^^,^^26^, ^27^, ^28^ but there may be a slight increase in revision surgery rate for open tenodesis.^20^ Arthroscopic approaches allow for concomitant arthroscopic procedures such as rotator cuff repair without converting to open dissection.^29^, ^30^, ^31^ We prefer the arthroscopic approach to avoid the risks of the open approach.

Options for tenodesis location include above or within the bicipital groove (arthroscopic), above the pectoralis major insertion below the bicipital groove (arthroscopic), or below the pectoralis major insertion (open). Although overall complication rates are low and some studies show no significant difference for tenodesis in the groove compared to those out of the groove, others have found that anchoring within or above the bicipital groove may be associated with continued pain.^32^^,^^33^ Furthermore, subpectoral approaches can risk damage to surrounding tissue and neurovascular structures. Fixation options include soft tissue tenodesis with suture or bony fixation using suture anchors or interference screws.^34^, ^35^, ^36^, ^37^, ^38^ Previous studies have found decreased clinical and structural outcomes with soft tissue tenodesis compared with the onlay and inlay bony fixation techniques.^39^ We prefer bony fixation below the groove performed through an arthroscopic technique to avoid the risk of groove pain while also negating the risks associated with the subpectoral approach.

LHBT anchoring can be achieved via inlay or onlay techniques.^39^^,^^40^ Bony fixation can be accomplished via interference screws,^41^ cortical buttons,^42^ or suture anchors.^43^ The inlay technique is noted for its superior biomechanical strength relative to the onlay technique.^44^ However, the inlay technique, which fixes the LHBT subcortically through an interference screw or a bicortical suspensory device, presents a potentially higher risk of fracture and a higher incidence of revision surgery,^45^, ^46^, ^47^ in part due to the risk of tendon tearing when entering bone at a sharp angle.^18^ The onlay technique lacks the structural integrity of the inlay technique, but it does carry a lower risk of Popeye deformity and affords a smaller footprint when compared with inlay anchors.^46^^,^^48^ Despite the shortcomings of each technique, studies show no significant difference in outcomes between onlay and inlay techniques.^19^^,^^46^^,^^49^ We chose an onlay technique to prevent the risk of fracture, “guillotine” rupture, and overtensioning of the LHBT, as well as to potentially expedite the procedure as there is no need to size the anchor to tendon diameter. With undertensioning of the LHBT, a persistent biceps deformity, early muscle fatigue, and cramping may occur, similar to that of tenotomy.^50^ With overtensioning of the LHBT, there is an increased risk of pullout at the tendon-screw-bone interface and fixation failure.^51^^,^^52^ By choosing an onlay fixation, we can improve our control of the tenodesis tension.

We outline our arthroscopic biceps tenodesis technique below the bicipital groove, above the pectoralis insertion, using an onlay suture anchor technique. We have found this technique to be highly reproducible and efficient, with the added benefit of minimal soft tissue disruption and risk of complications compared to open biceps tenodesis.

## Disclosures

The authors declare the following financial interests/personal relationships which may be considered as potential competing interests: M.B.G. receives institutional support from Skeletal Dynamics, 10.13039/100014388Acumed, and 10.13039/100007307Arthrex; receives research support from 10.13039/100008894Stryker and Konica Minolta; is a board or committee member of the American Society for Surgery of the Hand; and is an associate editor for the *Journal of Hand Surgery* and *Surgical Techniques in Orthopedics*. E.R.W. receives consulting fees from Stryker, Smith & Nephew, Depuy-Synthes, and Acumed, as well as institutional research support from Konica Minolta. All other authors (J.Z., Z.B.H., J.A.W., F.L.V., M.B.G.) declare that they have no known competing financial interests or personal relationships that could have appeared to influence the work reported in this paper.

## Supplementary Data

Video 1 Annotated procedure video of arthroscopic biceps tenodesis.
